# Embodied decision biases: individually stable across different tasks?

**DOI:** 10.1007/s00221-023-06591-z

**Published:** 2023-03-13

**Authors:** Eric Grießbach, Philipp Raßbach, Oliver Herbort, Rouwen Cañal-Bruland

**Affiliations:** 1 Department for the Psychology of Human Movement and Sport, Friedrich Schiller University Jena, Jena, Germany; 2 Department of Psychology, Julius-Maximilians-Universität Würzburg, Würzburg, Germany

**Keywords:** Interindividual differences, Decision-making, Motor control, Motor cost, Cognitive crosstalk, Embodied decisions, Bias

## Abstract

In everyday life, action and decision-making often run in parallel. Action-based models argue that action and decision-making strongly interact and, more specifically, that action can bias decision-making. This embodied decision bias is thought to originate from changes in motor costs and/or cognitive crosstalk. Recent research confirmed embodied decision biases for different tasks including walking and manual movements. Yet, whether such biases generalize within individuals across different tasks remains to be determined. To test this, we used two different decision-making tasks that have independently been shown to reliably produce embodied decision biases. In a within-participant design, participants performed two tasks in a counterbalanced fashion: (i) a walking paradigm for which it is known that motor costs systematically influence reward decisions, and (ii) a manual movement task in which motor costs and cognitive crosstalk have been shown to impact reward decisions. In both tasks, we successfully replicated the predicted embodied decision biases. However, there was no evidence that the strength of the biases correlated between tasks. Hence, our findings do not confirm that embodied decision biases transfer between tasks. Future research is needed to examine whether this lack of transfer may be due to different causes underlying the impact of motor costs on decisions and the impact of cognitive crosstalk or task-specific differences.

**Supplementary Information:**

The online version contains supplementary material available at 10.1007/s00221-023-06591-z.

## Introduction

In everyday life, we often make decisions during actions. For instance, while driving the car we decide to change the lane, when playing soccer, we decide to play the ball to a left or right-positioned teammate, or when walking through the shoe store we decide for which pair of shoes to stop. A commonality between these decision-making examples is that, first, actions are required to implement a decision and that, second, these actions are continuously changing over time, thereby qualifying these decisions as “embodied decisions” (Gordon et al. 2021). For example, while walking through the shoe store, your position in relation to the pair of shoes and accordingly the actions necessary to approach the shoes are constantly changing.

To account for embodied decisions, action-based models like the affordance competition hypothesis (Cisek 2007) and the embodied choice framework (Lepora and Pezzulo 2015) argue in favor of a bidirectional relationship between action and decision-making. That is, decisions not only influence subsequent actions in a hierarchical, top-down fashion (Cooper and Shallice 2000; Newell and Simon 1972), but actions also bias decision-making. In support of action-based models, a number of recent studies provided empirical evidence for this bidirectional relationship for various types of actions such as manual movements like reaching (Bakker et al. 2017; Cos et al. 2021; de Comité, Crevecoeur and Lefèvre 2022a, b; Michalski et al. 2020; Nashed et al. 2014; Pierrieau et al. 2021) or mouse tracking (Raßbach et al. 2021), and walking (Grießbach et al. 2021, 2022). Results from the latter three recent studies suggest that for both tasks, that is, walking and manual movements, the magnitude of the embodied decision biases strongly varies between participants (Grießbach et al. 2021, 2022; Raßbach et al. 2021). While some participants show no or only a small influence of concurrent action on decision-making, others are highly influenced by their concurrent actions.^1^ The observed interindividual differences prompt the question of whether embodied decision biases may generalize across tasks and hence be stable (i.e., trait-like) within individuals or whether embodied decision biases are task-specific.

On the one hand, there is initial evidence in favor of the generalization hypothesis. First, previously studied tasks such as manual movements and walking share certain properties including the selection of the speed of movement which tends to correlate within participants. For instance, it has been shown that people who reach faster also tend to walk faster (Labaune et al. 2020). Labaune and colleagues explain this relationship with a common control process for the selection of speed. Second, deciding while walking is essentially multitasking (Raßbach et al. 2021). In multitasking, the execution of two or more tasks is known to affect each other and the strength of these influences generalizes between tasks. This generalization is argued to reflect a trait-like multitasking ability (e.g., Morgan et al. 2013; Watson & Strayer 2010) or a stable individual preference for strategic task coordination (e.g., Bruning et al. 2021). Similarly, if the embodied decision bias was stable across tasks, it would point either to individually stable strategic preferences or a common higher-order control process. Furthermore, if embodied decision biases transfer between tasks, this might be practically useful to predict behavior, for instance, from a computerized task to behavior under more ecological conditions (like turning left or right while walking, driving a car, etc.). This may be particularly relevant in rehabilitation contexts (Marinho et al. 2019; Rowe and Siebner 2012), for the diagnoses of psychological disorders (Cohen and Verghese 2019), or for optimizing task performance (Anguera et al. 2013).

On the other hand, some findings speak in favor of the task-specificity of embodied decision biases. From a neurophysiological perspective, decisions and actions do not have a clear boundary. Decision-relevant information like the value of choice options blends together in effector-specific networks in the brain (Cisek and Kalaska 2010). That is, the neuronal activation for decisions effectuated with reaching movements is separately represented compared to eye movements or leg movements. If blending between action and decision plays a role in embodied decision biases, it is conceivable that such biases are effector-specific and hence variable between tasks.

**Fig. 1:**
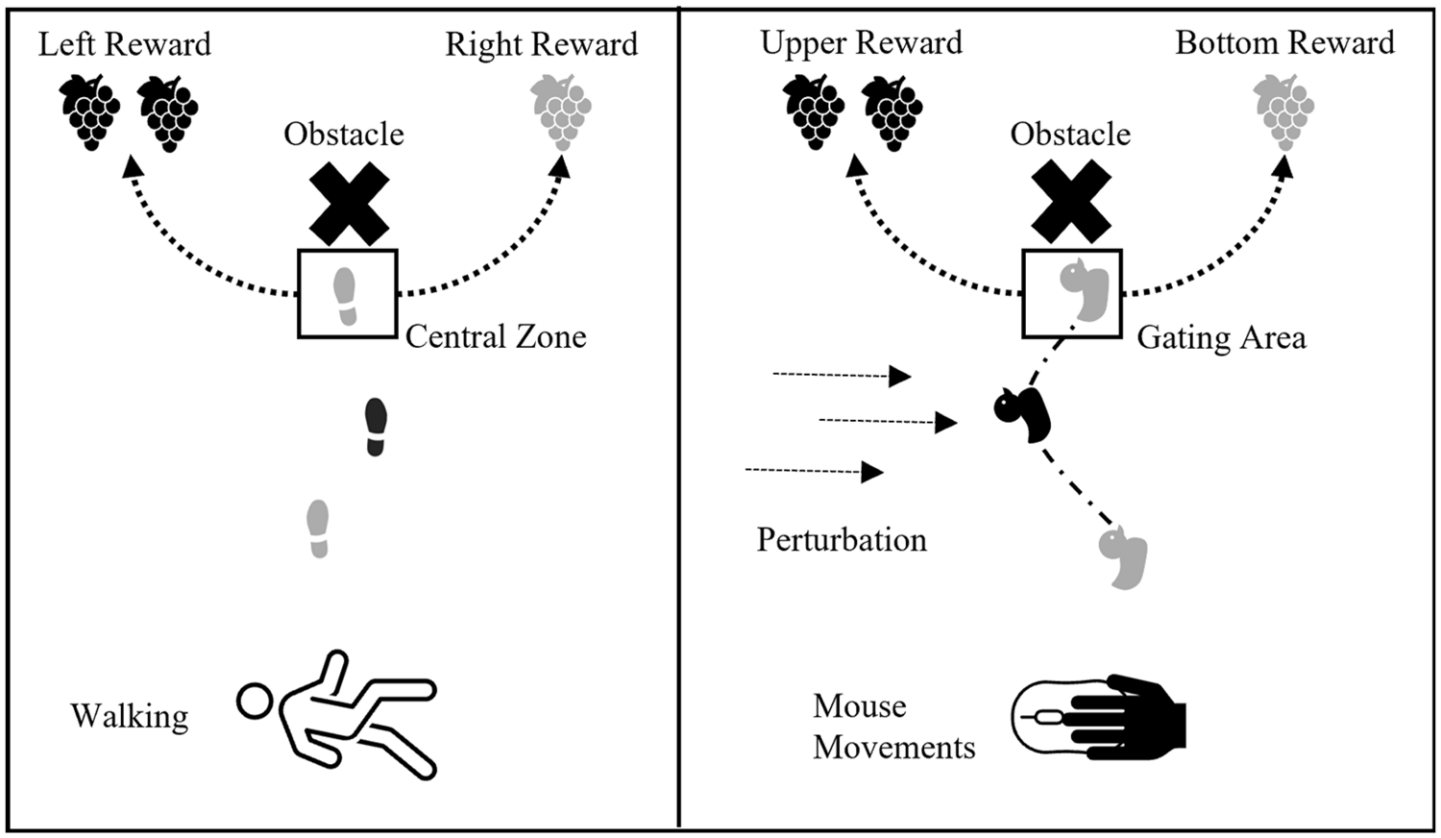
Conceptual representation of the experimental design of the walking task and the manual movement task. Lower action costs towards either choice option are exemplified by the same color coding between the current body state, i.e., swing leg in the walking task, vertical position of the bird in the manual movement task, and reward. **A** Walking task. **B** Manual movement task. Note that the display of the manual movement task is rotated 90° for clarity reasons. A more detailed presentation of both experiments is displayed in the supplement (fig. S1 and S2)

To investigate whether embodied decision biases generalize across different tasks within individuals, we asked participants to perform a manual movement task and a walking task (see Fig. 1). Both tasks have revealed embodied decision biases reliably (Grießbach et al. 2021, 2022; Raßbach et al. 2021). As illustrated in Fig. 1, in both paradigms, participants continuously move towards an obstacle that they have to pass by. Passing by the obstacle on one side or the other, however, yields different rewards. While in the walking paradigm (from now on referred to as TWWT, turning while walking task) participants walk toward the obstacle, in the manual movement task (from now on referred to as the MLTT, multilane tracking task) participants track a lane by controlling a cursor with the computer mouse. Applying these two tasks in a within-participant design, we tested the following general hypotheses: if embodied decision biases transfer between tasks, then the strength of the biases should correlate. If embodied decision biases are task-specific, then the strength of the biases should not correlate between tasks. In the following section, first, we further specify the tasks and provide more detail on how embodied decision biases are operationalized within the two tasks. We then translate the general hypotheses into specific, experimental hypotheses.

### The walking task (TWWT)

Action-based models argue that action information feeds back into the decision process (Lepora and Pezzulo 2015). Hence, it is possible to track action-related variables such as motor costs and weigh decision-making in real-time (de Comité, Lefèvre, and Crevecoeur 2022a; b; Wispinski et al. 2020), an assumption that has been backed up by various experimental studies (Brenner and Smeets 2015; Cos et al. 2021; de Comité, Crevecoeur, and Lefèvre 2022a, b; Marti-Marca et al. 2020). In the walking task, we focused on the embodied decision bias based on changes in motor costs (Grießbach et al. 2021, 2022). While walking, the motor cost to turn varies with the current swing leg. Turning towards the side of the current swing leg enables an easier lateral step. Turning the opposite of the current swing leg requires a more effortful cross-over step (Moraes et al. 2007; Patla et al. 1991; Taylor et al. 2005). To investigate the potential impact of motor costs on decision-making, the cost to turn in front an obstacle was manipulated by predetermining the starting position (e.g., left foot in front of the right foot) and thereby the side of the swing leg when turning. An embodied decision bias by means of motor costs would be reflected in a preference to choose the side enabling an easier lateral step. Indeed, the swing leg influenced decision-making, indicating an embodied decision bias due to changes in motor costs. Alternatively, the swing leg effect could also be based on representational overlap between decision-making and concurrent motor control (e.g., a shared representation between the left swing leg and left decisions), often observed in multitasking research (Hommel 1998; Janczyk et al. 2012, 2014). Using a computerized version of the walking task requiring manual movements, we aimed to disentangle this “cognitive crosstalk” and the bias by motor cost in a subsequent study.

### The manual movement task (MLTT)

In the MLTT, instead of walking participants had to track a horizontal lane with a virtual bird avatar (Raßbach et al. 2021). A constant downward or upward perturbation of the avatar required scrolling the mouse wheel up or down. Concurrently, a central obstacle moved toward the avatar and participants had to decide to switch to a parallel upper or lower lane offering different rewards. Instead of turning while walking, participants decided for a lane switch by moving the mouse forward or backward in the horizontal plane. Cognitive crosstalk varied based on the required scrolling direction to stabilize the avatar. The motor cost for a lane switch was manipulated by reversing the mapping between avatar position (and, thus, scrolling direction) and the necessary movement amplitude for a lane switch in different blocks.

Indeed, results showed that participants’ decisions were not only influenced by the required movement amplitude but additionally by the concurrent scrolling action to stabilize the avatar on the lane. That is, participants switched more often to the upper lane when scrolling upwards (moving the avatar upward) compared to downwards (moving the avatar downward). These s hence confirmed an embodied decision bias based on motor costs and additionally suggest cognitive crosstalk between action and decision-making.

### Experimental hypotheses

In sum, both in the walking task and in the manual movement task we found evidence for embodied decision biases. In the present study, we aimed at testing whether such biases generalize within individuals across different tasks. If these biases generalize across tasks, we expected a positive correlation between the swing leg effect (SLE) in the TWWT and the scrolling effect and/or cost effect in the MLTT. If these biases are task-specific, there should be no correlation between the SLE and the scrolling effect. Additionally, if the SLE in the walking task correlates with the scrolling effect, it would suggest that the SLE is partly driven by cognitive crosstalk, and not exclusively by motor costs.

## Methods

### Participants

We planned to do our power analysis by calculating Bayes factors (BF) and stop when BF_10_ > 10 or BF_10_ < 10 for both hypothesis-relevant correlation terms or when hitting 100 participants. We had to stop after 89 participants because of moving the lab to a new location. For eight participants there was a problem with saving the data for the MLTT and no data was available. They had to be excluded from the final analysis. Two participants always stepped with the same foot into the central zone, which meant that they would not contribute to the estimate of the SLE in the walking experiment. Therefore, they were also excluded from the analysis. After exclusion, the final sample size was *n* = 79 (mean age 22.7 years, SD = 3.5 years, 44 females, 35 males, 76 right-handed, 2 left-handed, 1 missing data for handedness, 66 right-footed, 8 no preference, 5 left-footed). Participants were compensated 15,00 € after the experiment, independent of performance. All participants gave informed consent before starting the experiment. Both experiments in the study were part of a research program that was approved by the ethics committee of the Faculty of Social and Behavioral Sciences of the Friedrich Schiller University Jena (FSV 19/04) and the ethics committee of the Department of Psychology of the University of Würzburg (GZEK 2019–33).

## Turning while walking task (TWWT)

### Apparatus and stimuli

The maximal distance from the starting line to the center of the central zone was 3.56 m (fig. S1). The left and the right targets were at a 52.5° angle at a 1.5 m distance from the central zone. The targets and the zone were 0.5 m × 0.5 m in size. To enforce a decision after reaching the central zone, three upright pipes (*r* = 3.7 cm, height = 55 cm) served as obstacles dividing the left and right sides after the central zone (60 cm behind the center of the central zone and with a 30-cm distance between obstacles). Black tape was used for the starting line, the central zone, and the lateral targets.

A digital projector displayed all stimuli (NEC Corp., Tokyo, Japan; Model M353WS, WXGA resolution, 60 Hz frame rate) on a large screen facing the participant (2.92 m width × 1.83 m height) at 1.80 m behind the center of the central zone. Black stimuli were presented on a white background. All stimuli were presented with a self-written script in MATLAB in real time based on the kinematic measurements (see data analysis). A 3D infrared system (12 cameras, Prime 17W, Optitrack, Corvallis, US) recorded Gait behavior (120 Hz) with passive reflective markers (12 mm) placed on the lateral malleolus, heel, and between the second and third metatarsal head of both feet.

Participants received auditory feedback indicating whether they finished in time after each trial. For auditory feedback served a beep (750 Hz for 0.8 s) or a double beep (750 Hz, two times 0.3 s with 0.2 s pause in between) with the integrated speaker of the projector and a sampling rate of 48,000 Hz. The meaning of the beep and double beep (in time or too late) was counterbalanced between participants. Individual time constraints and starting positions were determined by baseline walking behavior before the experiment (see supplement).

Stimuli were presented in real time based on the position of the marker. To do this, the 3D positions of markers were streamed with the NatNet SDK from Motive v2.1.1 (Optitrack software interface) to a self-written MATLAB 2018a script (The Mathworks, Inc., Natick, MA, USA). Important events included the start of a trial, the timing of reward presentation at the third step (Banks et al. 2015), the step in the central zone, and the end of a trial based (see supplement for further information about detection of these events).

### Procedure

The order of experiments was counterbalanced. Participants either started with the TWWT or the MLTT. Concerning the TWWT, participants started by providing informed consent and demographic data. Next, the instructor attached reflective markers on the feet. The experiment started with five baseline trials to determine the starting position and the time constraint (see supplement). Afterward, participants watched a narrated presentation of the instruction.

The instruction prompted them to collect rewards by walking toward one of two lateral targets. To start a trial, they had to position their feet into the predetermined starting position (left or right foot in front at the starting line) displayed on the screen. After 1.5 s, a central “ + ” appeared as the Go-Signal. After three steps one of three reward (point) combinations for the left and right targets appeared on the screen (left/right: 40/60, 50/50, or 60/40; participants have not been informed about the exact timing). Participants had to step into the central zone before walking towards a lateral target. To receive either, participants had to stand in the left or right target with both feet. Afterward, auditory feedback signaled the time information. If they were in time, they received the reward of the chosen side. If they were too late, they received the lower reward (40 points if 60/40, 50 points if 50/50). After the trial, participants could start the next trial on their own.

Each participant completed a total of 12 familiarization trials and 60 experimental trials. The experimental phase included 2 (starting position: left/right foot at starting line) × 3 (point combination left/right: 40/60, 50/50, 60/40) × 10 trials. Trials were presented in random order. The task lasted about 30 min.

## Multilane-tracking-task (MLTT)

### Apparatus and stimuli

The MLTT was conducted in a separate room. Participants were seated 60 cm in front of a LED monitor (ASUS TUF Gaming VG259QM) with a screen diagonal of 24.5 inches, a screen resolution of 1920 by 1080 pixels, and a refresh rate of 120 Hz. The main input device for the experiment was a Fujitsu M530 computer mouse (1200 dpi) connected via USB to the lab PC. The experiment was realized with a self-written Python script (version 3.7), mainly using the Python module *pygame* (Shinners 2011). The basic visual scenery of the MLTT consisted of three horizontal white lanes on a black background spanning the entire display (visual angle per lane: 3.63°). The bird avatar (height and width in degree visual angle: 2.42°) was displayed as a schematic yellow bird (see fig. S2). Two obstacles (depictions of cats) were positioned on the upper and lower lane and represented a gate (height and width in degree visual angle per obstacle: 3.63°; horizontal position: 1.13 × 1920 px). One similar obstacle was positioned on the middle lane and represented the obstacle participants had to evade, comparable to the pipes in the TWWT (horizontal position: 0.92 × 1920 px). Rewards were displayed as yellow stars on the upper and lower lane (height and width in degree visual angle: 3.02°; horizontal position: 1.35 × 1920 px) as well as numerals in white text (Arial, font size 31 pts [1.04° visual angle]) to the right above and below the bird for the upper and lower star, respectively (see fig. S2). Importantly, only the numerals were informative about the points associated with the stars. The reward objects always had the same size and only signaled the end of a trial.

Movement of the visual scenery was realized by shifting the obstacle and reward stimuli to the left of the screen with an average movement speed of about 5 pixels per frame update. A frame update was performed every 10 ms and the current state of a trial was recorded after each update (i.e., the sampling rate was 100 Hz). Frame rate and program logic were decoupled so that frame time deviances did not profoundly impact temporal stimulus events such as collisions or reward presentation. The horizontal position of the bird was fixed while its vertical position was determined by a unidirectional perturbation upward or downward, uniformly randomized between roughly 9 and 14 pixels, applied every 100 ms.

The motor cost manipulation, explained in more detail in the following Procedure (MLTT) section, was implemented by varying the mouse movement threshold for a lane switch as a linear function of the bird's position on the middle lane (see supplement). A visual cue (bird rotating and pointing in the direction of lower motor costs, see fig. S2) was implemented to facilitate motor cost integration.

### Procedure

Participants first received written instructions about the MLTT on the computer screen. They were informed that they would control a small yellow bird moving from left to right across one of three horizontal lanes. The instructions also noted that the bird would be vertically perturbed by gusts of wind (i.e., the perturbation), pushing the bird either upward or downward within a trial. To prevent the bird from drifting too far from the lane, participants had to scroll downwards or upwards with the mouse wheel to move the bird back to the center of the lane. Participants were also informed that several objects (obstacles and rewards) would appear within a trial. They were told to evade the central obstacle by performing mouse movements to switch lane (forward to the upper lane and backward to the lower lane). The instructions also noted that the necessary movement amplitude for lane switches would vary as a function of the bird's position on the middle lane in the different experimental blocks and that the rotation of the bird avatar would indicate the direction of higher/lower action costs. Most importantly, participants were instructed to accumulate as many points as possible by collecting stars.

After the general instructions, participants were instructed and could practice the MLTT in one block of 30 trials for each the *congruent* and *incongruent* motor cost condition (60 trials in total). In the *congruent* condition, if the bird was perturbed upward and was thus, on average, positioned on the upper half of the middle lane, switches to the upper lane required a shorter mouse movement but a longer movement in the *incongruent* position dependence condition (see fig. S2). Conversely, if the cursor was perturbed downward and was positioned on the lower half of the middle lane, switches to the upper lane required a shorter mouse movement in the *incongruent* but a larger amplitude mouse movement in the *congruent* condition.

After the practice trials, the experimental trials followed. Each trial started with a 1000 ms long display of the stationary visual scene including the lanes and the bird. Participants were instructed to use this time interval to reset the computer mouse position from the preceding trial to a neutral starting position for the next trial. Afterward, the scene shifted to the left and the perturbation started to push the bird either upward or downward. If participants did not counteract the perturbation and consequently drifted too far from the currently tracked lane (i.e., the center of the bird outside the bounds of 425 and 655 pixels on the vertical axis of the screen), a corresponding error message was displayed (“Der Vogel wurde von der Bahn geweht!”, which is German for “The bird was blown off the track!”). After 3250 ms of performing the motor control task, the obstacles and rewards were displayed. Participants then had 750 ms to perform a mouse movement forward or backward to switch to the upper or lower lane, respectively. If participants failed to perform a mouse movement of sufficient amplitude for either lane switch direction within the 750 ms interval, they collided with the central obstacle, and an error message was displayed (“Oh nein, die Katze auf der mittleren Bahn hat Dich gefressen!”, which is German for “Oh no, the cat on the middle lane has eaten you!”). In case of a successful lane switch, the cursor instantaneously moved to the respective lane and participants still had to counteract the perturbation until the bird reached the reward object (star). Then, the next trial started. Each trial had a duration of 5000 ms, with error trials having a duration of 6000 ms.

Participants worked on 2 (position dependence of motor costs: *congruent*, *incongruent*) × 2 (perturbation: *upward*, *downward*) × 3 (point combination upper/lower lane: 40/60, 50/50, 60/40) × 20 (repetitions) experimental trials (240 trials in total). Position dependence of motor costs was manipulated blockwise with block order being randomized for each participant separately. All other factors were manipulated trial wise with trial order being randomized for each block and participant separately. The MLTT part of the experiment lasted about 40 min.

### Data analysis

In the TWWT, kinematic data were filtered at 12 Hz with a bidirectional fourth-order low-pass Butterworth filter. We interpolated missing values up to 25 frames (0.21 s, cubic spline interpolation). 5100/5265 trials (97%) were included in the statistical analysis. Trials were excluded because participants made only three steps until reaching the zone and hence rewards were displayed too late (68 trials) or because of problems with the measurement (97 trials, losing a marker while walking, or tracking problems).

In the MLTT, 19700/20336 (97%) trials were included in the final analysis. 636 trials were excluded because participants did not perform a clear lane switch movement with the mouse in between the gating zone. We defined a lane switch movement as movements during the trial exceeding 50 pixels which was roughly the cut-off for differences in the distribution for movement lengths around zero compared to peaks below or above zero as observed in a histogram.

For statistical analysis, we used R (R Core Team 2019). To calculate estimates of the embodied decision biases on decision-making both in the MLTT and the TWWT together with the correlation of these effects we used a bayesian multivariate logistic mixed regression model for our statistical analysis. Multivariate models consider the dependencies of multiple dependent variables, which in our case were the respective choices in both the MLTT and the TWWT. Mixed models can take repeated measures (dependent measures) of participants into account (Brauer and Curtin 2018). More specifically, this is achieved by including random effect parameters which give an estimate of the variance between participants. Mixed models simultaneously allow to include correlation estimates of the variance between participants, which indicate, for example, whether participants with a high/low effect size in the SLE also have a high/low effect size in the scrolling effect.

Since our outcome variable “decision” was in both experiments restricted to left/right or up/down, we used a binomial distribution for participants choices. To linearize the model, we used a logit link function. Finally, multiple reasons led us to fit the model under a Bayesian Framework. Firstly, generalized mixed models in a frequentist framework suffer from convergence problems (Eager and Roy 2017). Secondly, we lacked the necessary data for conducting a power analysis, so we adopted a Bayesian approach for our sampling approach instead, which does not require this information. Third, Bayesian approaches provide some benefits in quantifying uncertainty, including the testing of evidence for the Null-hypothesis and not only against it (Wagenmakers et al. 2016).

For the decision in the TWWT, we included the *swing leg* as a predictor. For the decision in the MLTT, we used *position dependence*, the inverse of *perturbation direction* (i.e., the presumed *scrolling direction)*, and their interaction as predictors. We used a priori specified contrasts based on our hypothesis (Schad et al. 2020). For the swing leg, position dependence, and scrolling direction, we used a centered sum contrast to compare the effect of the right swing leg/congruent/downwards (− 0.5) vs. the left swing leg/incongruent/upwards (+ 0.5).

We included all random effects (two variance parameters for the intercepts, four for the slopes, and fifteen correlation parameters) in the model. In prior studies, embodied decision biases were observed for all reward combinations, even though partially in different magnitudes (Grießbach et al. 2021; Raßbach et al. 2021). Hence, we decided not to include reward combination as a predictor as the number of estimates for the model would increase drastically (Brauer and Curtin 2018). Model fitting was done with the brms package (Bürkner 2017). We followed the guidelines of Kruschke (2021) for the statistical analysis. Bayesian models allow to include prior distribution for all estimates. For all priors we used weakly informative priors which serve as a regularization of unrealistic values to reduce the risk of overfitting. The priors are specified in the SI and script. Our script are publicly available at https://osf.io/8gxqe/?view_only=5cd1d6bf48e84e30a3096f36354ffc0d.

For each parameter, the Bayesian model provides a posterior distribution. The posterior distribution is a probabilistic representation of parameter values given the priors, the likelihood of the data, and the model. To summarize the posterior distribution, we provided the estimated mean ($\widehat{\beta }$), the equal-tailed 95% credible interval (CrI), and the probability for samples below or over a certain value. The 95% CrI defines the range within which the parameter value falls with a probability of 95%. We highlight parameters for which over 95% of the posterior distribution (values) are positive/negative compared to negative/positive below in the text.

We also provide Bayes Factors as a measure of whether data shifted the likelihood towards or away from the null model (*β* = 0) compared to the prior likelihood, suggesting a change in evidence for or against the null model. As our hypotheses are directional, we expect a positive correlation. We also tested the evidence ratio of the effect being positive compared to a negative correlation.

## Results

### Reward influenced decision-making

Before examining the influence of action on decision-making, we validated whether participants followed the instruction by fitting a model only with rewards as a predictor for target decisions. Reward influenced participants' decisions in both tasks. For the TWWT, participants went more often to the right side given that 60 points were displayed on the right side versus 60 points on the left side (OR = 38.07, 95% CrI = 21.94 to 70.35, *p*(OR > 1) > 0.999, BF_01_ < 0.001). The same was true for the MLTT (OR = 1.66, 95% CrI = 1.39 to 2.00, *p*(OR > 1) = 0.999) > 0.99, BF_01_ < 0.001).

The influence of reward on decision-making correlated between experiments (*r* = 0.37, 95% CrI = 0.12 to 0.60, *p*(*r* = 0) = 0.05, BF_01_ = 0.06, *p*(*r* > 0) > 0.999, RE_+_  = 340.23). That is participants who accumulated more rewards in the MLTT also accumulated more rewards in the TWWT.

### Concurrent action influenced decision-making

**Fig. 2:**
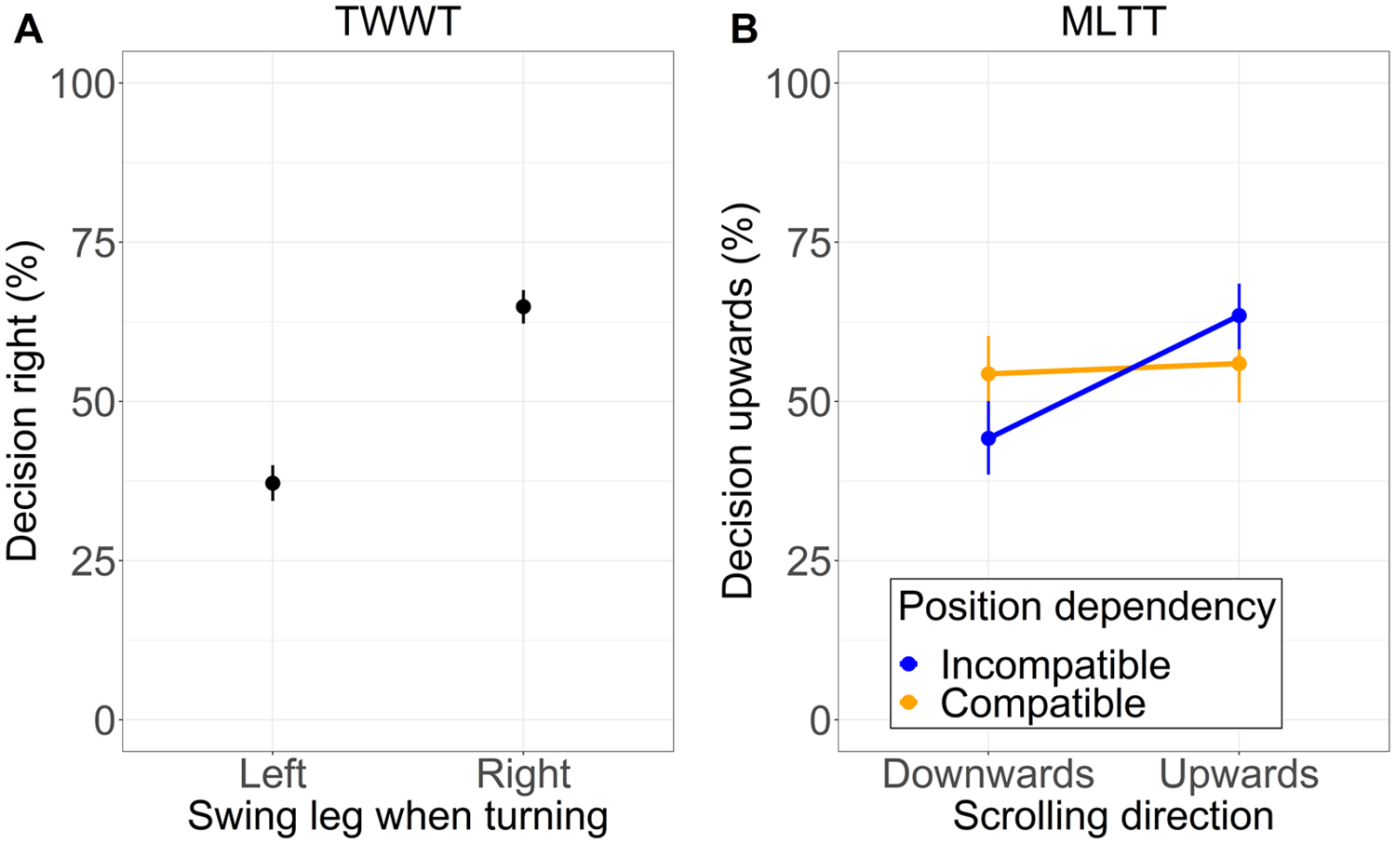
Swing leg effect in the TWWT, scrolling effect, and cost effect in the MLTT. Estimates are marginalized over reward feedback. The SLE is defined by the preference to turn towards the side of the swing leg (left side when turning with a left swing leg and vice versa). The scrolling effect is the preference to jump toward the direction of scrolling with the mouse wheel. The cost effect is based on the interaction of the scrolling direction and the position dependence condition

Table 1 summarizes the posterior distributions of the correlation model. Figure 2 displays the probability scales of the individual effects. Odds ratios below 1 correspond to a higher likelihood for a leftwards (TWWT)/downwards (MLTT) choice and odds ratios greater than 1 for a rightwards (TWWT)/upwards (MLTT) choice. As displayed in Table 1, embodied decision biases were present in both tasks. In the TWWT, participants preferred walking towards the right side given a right swing leg compared to a left swing leg (SLE, Fig. 2A). In the MLTT, participants preferred to switch upwards given that they had to compensate for a downwards perturbation by scrolling upward (scrolling main effect, Table 1 and Fig. 2B). Additionally, the scrolling effect interacted with the position dependence (cost effect, Table 1 and Fig. 2B).

**Table 1:** Parameter summary of the fixed effects

Effect	OR	95% CrI	P(OR) < 1	BF_01_
TWWT: intercept	1.05	[0.98–1.12]	0.10	–
MLTT: intercept	1.20	[1.02–1.41]	0.01	–
TWWT: SLE (left:right)	**3.13**	**[2.57–3.80]**	** < 0.001**	** < 0.01**
MLTT: dependency (comp:incomp)	0.95	[0.88–1.03]	0.89	6.18
MLTT: scrolling effect (downwards:upwards)	**1.53**	**[1.20–1.95]**	** < 0.001**	**0.01**
MLTT: cost effect (dependency:scrolling effect)	**2.06**	**[1.26–3.34]**	**0.002**	**0.03**

Each parameter is summarized as the mean odds ratio, the 95% credible interval, and the probability that the posterior is smaller than one. The hypothesis-relevant parameters are marked in bold. For contrasts see methods

### No correlation of embodied decision biases between tasks

**Fig. 3:**
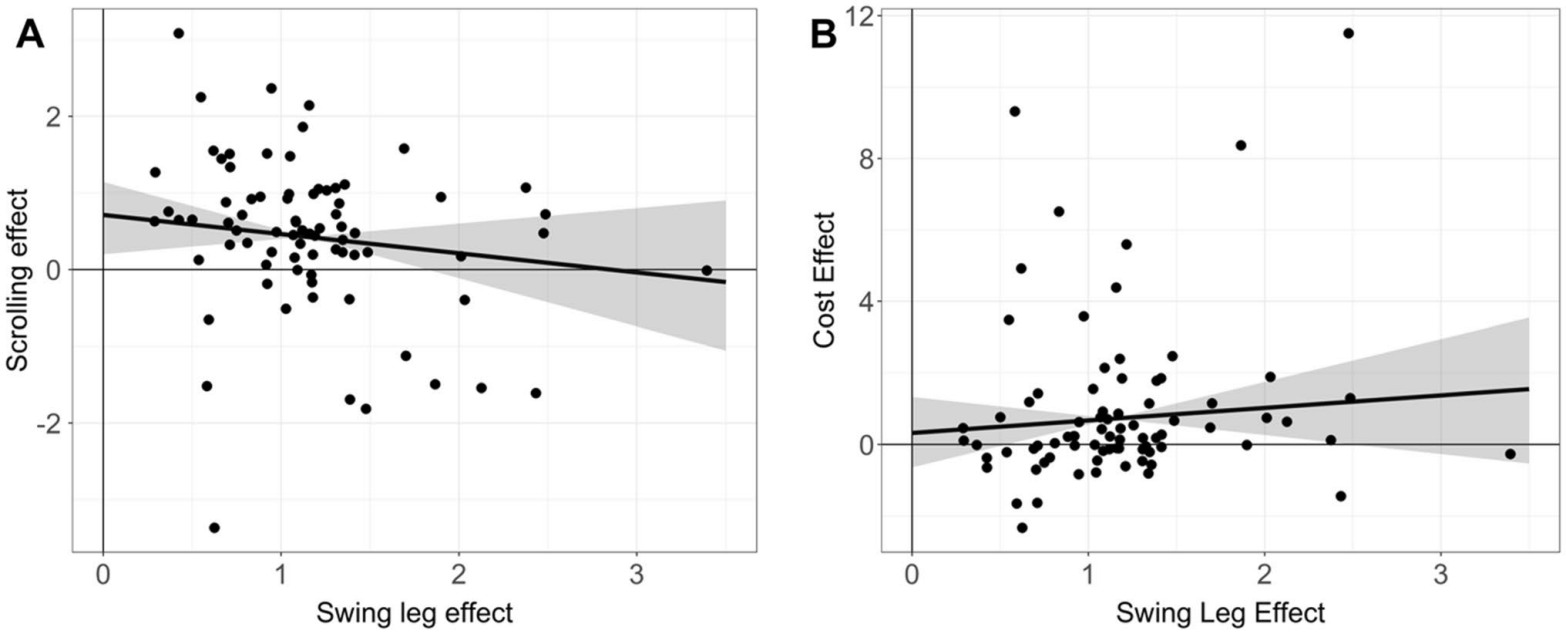
Correlation between the effects of both tasks. **A** Scatter plot between the scrolling effect and the SLE. **B** Scatter plot between the SLE and the cost effect. Displayed are the estimates of random effects of individual participants as individual points (note that these are shrinked towards the mean, a property of mixed models), the mean correlation line (note that the correlation line is not based on the individual estimates per se) and 95% CrI. The 95% CrI is 0 at the means of both effect because only variation in the slope is plotted and not variation in the intercept (mean was taken). Data is displayed as log odds ratios

Next, we focused on the correlation term between the random slopes for the TWWT and the MLTT (see Fig. 3).

The SLE and the scrolling effect were not positively correlated, but the correlation was negative or near zero, *ρ* =  − 0.16 (95% CrI =  − 0.42 to 0.11, BF_01_ = 1.13, *p*(*ρ* = 0) = 0.53). More specifically, the Bayes Factor near 1 indicated that it is inconclusive whether the null hypothesis (*ρ* = 0) or the alternative hypothesis is preferred (*ρ* ≠ 0). However, our hypothesis explicitly states a positive correlation between the SLE and the scrolling effect. Hence, we additionally calculated the evidence ratio of the slope being positive rather than negative (ER_+_  = 0.13, *p*(*ρ* > 0) = 0.12). The Evidence Ratio below 1 indicates moderate evidence that the correlation between the SLE and the scrolling effect is negative.

Concerning the SLE and the motor cost effect in the MLTT, if there was a positive correlation between both effects, it would be weak at most. More specifically, the correlation between the SLE in the TWWT and the motor cost effect in the MLTT is estimated to be *ρ* = 0.10 (95% CrI =  − 0.16 to 0.36, BF_01_ = 1.74, *p*(*ρ* = 0) = 0.63). Again, the Bayes Factor near 1 indicates that it is inconclusive whether the null hypothesis (*ρ* = 0) or the alternative hypothesis is preferred (*ρ* ≠ 0). However, our hypothesis explicitly states a positive correlation between the SLE and the motor cost effect. Hence, we additionally calculated the evidence ratio of the slope which was positive rather than negative (ER_+_  = 3.54, *p*(*ρ* > 0) = 0.78), indicating moderate evidence that the correlation between the SLE and the motor cost effect is positive. However, even if there would be a positive relationship, this relationship is likely to be small (e.g., for *ρ* > 0.32: ER_>0.32_ = 0.05, *p*(*ρ* > 0.32) = 0.05). Additionally, this effect is highly dependent on one participant (see Fig. 3B). After exclusion of this participant, the correlation decreased and averaged into a negative value (*ρ* =  − 0.04, 95% CrI =  − 0.27 to 0.19, ER_+_  = 0.63, *p*(*ρ* > 0) = 0.39).

In conclusion, the correlations between the embodied decision biases observed in each task are negative, or close to zero. Hence these results do not indicate that these biases are strongly positively correlated between tasks.

### Reliability of measures

To test whether the embodied decision biases are stable measures and suited for the analysis of individual differences, we tested the reliability of the measures with a split-half analysis. That is, we assigned every second trial to one of two levels to compare whether the effect correlates between these trials (Schuch et al. 2021). The split-half correlation for the SLE in the TWWT was *r* = 0.74 (95% CI = 0.39 to 0.95, BF_01_ < 0.01, *p*(*β* = 0) < 0.001), and the split-half correlation for the scrolling effect in the MLTT was *r* = 0.93 (95% CI = 0.85 to 0.98, BF_01_ < 0.01, *p*(*β* = 0) < 0.001). For the cost effect in the MLTT, the split-half correlation was *r* = 0.95 (95% CI = 0.90 to 0.89, BF_01_ < 0.01, *p*(*β* = 0) < 0.001). Hence, the reliability of all three effects was high and the embodied decision biases are suitable for the analysis of interindividual correlations.

## Discussion

In this study, we aimed to examine whether embodied decision biases generalize across tasks such as walking and manual movements (Bruning et al. 2021; Harter and Leahy 1999; Labaune et al. 2020; Morgan et al. 2013) or whether they are task-specific (Cisek and Kalaska 2010). To this end, participants performed two tasks that reliably produced embodied decision biases in previous studies but differed in the concurrent task requirements, namely, a walking task (Grießbach et al. 2021), and a computerized version of the walking task requiring manual movements (Raßbach et al. 2021). We predicted that if embodied decision biases transfer between tasks, then the size of the biases in the walking and the manual movement task should correlate. By contrast, if embodied decision biases are task-specific, then the size of the bias should not correlate between tasks. Results showed that both tasks separately produced the predicted embodied decision biases, thereby replicating the findings of previous studies (Grießbach et al. 2021, 2022; Raßbach et al. 2021). However, concerning the main research question, results did not reveal a positive correlation between embodied decision biases of the two distinct tasks. Yet, the impact of reward was correlated between tasks, that is, participants who received more rewards in the TWWT also received more rewards in the MLTT. Each of these findings will be discussed in detail in the remainder of the discussion.

### Embodied decision biases in the TWWT and the MLTT

We were able to replicate embodied decision biases in both tasks. In the TWWT, participants' decisions to turn while walking was influenced by the side of the alternating swing leg when turning. As the motor cost changes based on the current swing leg (Patla et al. 1991; Taylor et al. 2005) the SLE suggests that action influences decision-making by dynamic changes in motor cost (Grießbach et al. 2021, 2022). Alternatively, the SLE could also be based on cognitive crosstalk (e.g., a shared representation between the left swing leg and left decisions), often observed in multitasking research (Hommel 1998; Janczyk et al. 2012, 2014).

The MLTT was designed to disentangle whether the embodied decision bias is driven by motor cost and/or cognitive crosstalk. In the MLTT, participants' decisions to switch to the upper or lower lane were influenced by the concurrent scrolling movements with the mouse wheel to counteract a perturbation of the bird avatar (indicating cognitive crosstalk) as well as by the mapping between avatar position (as predominantly determined by the perturbation) and the required movement amplitude for lane switches in either direction (indicating a motor cost bias). Hence, the results from both tasks corroborate the claim from action-based models that the decision process and action are heavily intertwined (Cisek 2007; Cisek and Kalaska 2010; Gordon et al. 2021; Lepora and Pezzulo 2015), and further add to the growing evidence showing that concurrent action influences the decision process (Cos et al. 2021; Grießbach et al. 2021; Pierrieau et al. 2021; Raßbach et al. 2021).

### Embodied decision biases did not transfer between tasks

Although we replicated embodied decision biases in both tasks, there was no evidence that these biases were positively correlated between tasks, neither for the SLE and the scrolling effect nor for the SLE and the cost effect in the MLTT. If anything, there were tendencies for a negative correlation between the SLE and the scrolling effect. There are several potential explanations for the non-existent (positive) correlation of embodied decision biases between tasks.

First, the lack of a correlation could be interpreted to indicate that the biases are rather task-specific and hence not stable across tasks within individuals. If true, this would mean, on the one hand, that embodied decision biases are not a result of a general multitasking ability (Morgan et al. 2013; Watson and Strayer 2010), stable individual preferences for task coordination (Bruning et al. 2021), or a common motor control process as reported for vigor (Labaune et al. 2020). On the other hand, it would raise the question what the task-specific features are that account for the differences in embodied decision biases within individuals across tasks. There are several candidates, that is, notable differences between the tasks which could make the crosstalk task-specific. First, because decision-relevant variables are reflected in effector-specific networks in the brain (Cisek and Kalaska 2010) and the effectors to implement the tasks differ, it follows that this difference in effectors may account for whether crosstalk generalizes between tasks or not (De Jong et al. 1995). In other words, the neural activations for reaching movements, eye movements, and leg movements are each separately represented (but for spatial crosstalk between eye and manual movements see Huestegge and Koch 2009). It is hence conceivable that embodied decision biases in a manual movement task such as the MLTT may only generalize to tasks that rely on arm movements, but not to walking tasks such as the TWWT. It may even be the case that embodied decision biases are not only effector-specific, but even movement- or action-specific. To further disentangle these possibilities, future studies are needed that manipulate the similarity of effectors and/or movements when studying interindividual differences of embodied decision biases between tasks.

Second, concerning cognitive crosstalk, the direction of movement was different between the tasks (i.e., left and right for the TWWT and upwards and downwards for the MLTT). If cognitive crosstalk is based on abstract representations of action (effects) like spatial similarity (Simon et al. 1970), it might be useful to compare tasks with spatially similar representations in future studies (e.g., left and right in both tasks). Importantly, while cognitive crosstalk may (at least partially) explain embodied decision biases in both the TWWT and the MLTT, it was only experimentally tested in the MLTT. Whether cognitive crosstalk also accounts for embodied decision biases in the TWWT remains to be determined.

Concerning the task-specificity of embodied decision bias driven by motor costs such as in the SLE, a relevant factor could be the actual and/or perceived motor cost varying between individuals. Motor costs of movements are reduced with familiarity and learning (Huang et al. 2012). If true, participants who are more skilled in making crossover steps in the TWWT (e.g., due to experience with such movements from playing soccer) may have a lower cost of making the cross-over step independent from the energetic cost for manual movements in the MLTT. Individual differences in the weight distribution could also become important for objectively different costs between both tasks. Hence, the cost itself becomes task-specific. Based on this argument, future studies would be well-advised to measure the motor cost more objectively and make them comparable between tasks. This could be done for example by measuring the absolute metabolic cost (Huang et al. 2012; McNarry et al. 2017) or relative force requirement (Morel et al. 2017) of movements.

Alternatively, it could be that the correlation between the effects of both tasks is rather small and is not reliably detectable with a sample size of *n* = 79 (Cohen 1992; Schönbrodt and Perugini 2013).^2^ This is especially true if the SLE, scrolling effect, or cost effect is unreliable, making a correlation harder to detect (Schuch et al. 2021). When testing for reliability with the split-half method, however, we observed that the SLE (*r* = 0.74), the cost effect (*r* = 0.95), and the cognitive crosstalk effect (*r* = 0.93) are relatively stable. Hence, a correlational approach between the effects of both tasks is justifiable. Nonetheless, split-half methods have their limitations, and reliability would be better solved by measuring the embodied decision biases across multiple sessions and analyzing whether the strength of these biases remains stable within subjects between sessions (Schuch et al. 2021).

Finally, our results showed that the impact of reward was positively correlated between tasks. Participants receiving more rewards in the TWWT also received more rewards in the MLTT. It suggests a common mechanism for the influence of reward between both tasks that is worth to be studied in more in-depth in future research. For instance, an overarching motivational component like a subjective sensitivity for rewards (e.g., Crane et al. 2018) might explain the stable, but inter-individually different impact of reward. Next to an effect of reward, it is conceivable that other factors such as control strategies shared between tasks may be at play too. These strategies might, for instance, include adaptations of lower-level control (Grießbach et al. 2021, 2022), which may influence performance also in other tasks like the MLTT (e.g., by adapting the state of the bird with scrolling action to buy time during decision-making). Additionally, participants could be prone to take longer to evaluate or even postpone making a final decision with the result being a change in speed-accuracy trade-offs, which seems to be relevant not only for decision-making but also for motor control (Spieser et al. 2017; Du et al.. 2022).

To conclude, we successfully replicated embodied decision biases in a walking task and a manual movement task. However, these biases did not generalize across tasks within individuals, suggesting that embodied decision biases are rather task-specific.

## Supplementary Information

Below is the link to the electronic supplementary material.

Supplementary file1 (PDF 461 KB)

## Data Availability

The data is available at: https://osf.io/8gxqe/?view_only=5cd1d6bf48e84e30a3096f36354ffc0d.

## Footnotes

1 For instance, with respect to manual movements see the variance (random effects) in Table 1 in Raßbach et al. (2021), or for walking see Table S1 to Table S4 in Grießbach et al. (2021).

2 E.g., from a frequentist perspective n = 85 would be needed to find a correlation of 0.3 with 80% power.

## Code availability

Custom codes for the statistical analysis are available at: https://osf.io/8gxqe/?view_only=5cd1d6bf48e84e30a3096f36354ffc0d.

## References

[CR1] Anguera JA, Boccanfuso J, Rintoul JL, Al-Hashimi O, Faraji F, Janowich J, Gazzaley A (2013). Video game training enhances cognitive control in older adults. Nature.

[CR2] Bakker RS, Weijer RHA, van Beers RJ, Selen LPJ, Medendorp WP (2017). Decisions in motion: passive body acceleration modulates hand choice. J Neurophysiol.

[CR3] Banks JJ, Chang WR, Xu X, Chang CC (2015). Using horizontal heel displacement to identify heel strike instants in normal gait. Gait Posture.

[CR4] Brauer M, Curtin JJ (2018). Linear mixed-effects models and the analysis of nonindependent data: a unified framework to analyze categorical and continuous independent variables that vary within-subjects and/or within-items. Psychol Methods.

[CR5] Brenner E, Smeets JB (2015). Quickly making the correct choice. Vision Res.

[CR6] Bruning J, Reissland J, Manzey D (2021). Individual preferences for task coordination strategies in multitasking: exploring the link between preferred modes of processing and strategies of response organization. Psychol Res.

[CR7] Bürkner PC (2017). brms: an R package for Bayesian Multilevel Models Using Stan. J Stat Soft.

[CR8] Cisek P (2007). Cortical mechanisms of action selection: the affordance competition hypothesis. Philos Trans R Soc Lond B Biol Sci.

[CR9] Cisek P, Kalaska JF (2010). Neural mechanisms for interacting with a world full of action choices. Annu Rev Neurosci.

[CR10] Cohen J (1992). A power primer. Psychol Bull.

[CR11] Cohen JA, Verghese J (2019). Gait and dementia. Handb Clin Neurol.

[CR12] Cooper R, Shallice T (2000). Contention scheduling and the control of routine activities. Cogn Neuropsychol.

[CR13] Cos I, Pezzulo G, Cisek P (2021). Changes of mind after movement onset depend on the state of the motor system. eNeuro.

[CR14] Crane NA, Gorka SM, Weafer J, Langenecker SA, de Wit H, Phan KL (2018). Neural activation to monetary reward is associated with amphetamine reward sensitivity. Neuropsychopharmacology.

[CR15] De Comite A, Crevecoeur F, Lefèvre P (2022). Reward-dependent selection of feedback gains impacts rapid motor decisions. eNeuro.

[CR16] De Comite A, Lefèvre P, Crevecoeur F (2022). Continuous monitoring of cost-to-go for flexible reaching control and online decisions. bioRxiv.

[CR17] De Jong R, Coles MGH, Logan GD (1995). Strategies and mechanisms in nonselective and selective inhibitory motor control. J Exp Psychol Hum Percept Perform.

[CR18] Du Y, Krakauer JW, Haith AM (2022). The relationship between habits and motor skills in humans. Trends Cogn Sci.

[CR19] Eager C, Roy J (2017). Mixed effects models are sometimes terrible. arXiv..

[CR20] Gordon J, Maselli A, Lancia GL, Thiery T, Cisek P, Pezzulo G (2021). The road towards understanding embodied decisions. Neurosci Biobehav Rev.

[CR21] Grießbach E, Incagli F, Herbort O, Cañal-Bruland R (2021). Body dynamics of gait affect value-based decisions. Sci Rep.

[CR22] Grießbach E, Raßbach P, Herbort O, Canal-Bruland R (2022). Embodied decisions during walking. J Neurophysiol.

[CR23] Harter S, Leahy RL (1999). The construction of the self: a developmental perspective. J Cogn Psychother.

[CR24] Hommel B (1998). Automatic stimulus-response translation in dual-task performance. J Exp Psychol Hum Percept Perform.

[CR25] Huang HJ, Kram R, Ahmed AA (2012). Reduction of metabolic cost during motor learning of arm reaching dynamics. J Neurosci.

[CR26] Huestegge L, Koch I (2009). Dual-task crosstalk between saccades and manual responses. J Exp Psychol Hum Percept Perform.

[CR27] Janczyk M, Pfister R, Crognale MA, Kunde W (2012). Effective rotations: action effects determine the interplay of mental and manual rotations. Journal of Experimental Psychology-General.

[CR28] Janczyk M, Pfister R, Hommel B, Kunde W (2014). Who is talking in backward crosstalk? Disentangling response- from goal-conflict in dual-task performance. Cognition.

[CR29] Kruschke JK (2021). Bayesian analysis reporting guidelines. Nat Hum Behav.

[CR30] Labaune O, Deroche T, Teulier C, Berret B (2020). Vigor of reaching, walking, and gazing movements: on the consistency of interindividual differences. J Neurophysiol.

[CR31] Lepora NF, Pezzulo G (2015). Embodied choice: how action influences perceptual decision making. PLoS Comput Biol.

[CR32] Marinho V, Pinto GR, Bandeira J, Oliveira T, Carvalho V, Rocha K, Teixeira S (2019). Impaired decision-making and time perception in individuals with stroke: behavioral and neural correlates. Rev Neurol.

[CR33] Marti-Marca A, Deco G, Cos I (2020). Visual-reward driven changes of movement during action execution. Sci Rep.

[CR34] McNarry MA, Wilson RP, Holton MD, Griffiths IW, Mackintosh KA (2017). Investigating the relationship between energy expenditure, walking speed and angle of turning in humans. PLoS ONE.

[CR35] Michalski J, Green AM, Cisek P (2020). Reaching decisions during ongoing movements. J Neurophysiol.

[CR36] Moraes R, Allard F, Patla AE (2007). Validating determinants for an alternate foot placement selection algorithm during human locomotion in cluttered terrain. J Neurophysiol.

[CR37] Morel P, Ulbrich P, Gail A (2017). What makes a reach movement effortful? Physical effort discounting supports common minimization principles in decision making and motor control. PLoS Biol.

[CR38] Morgan B, D'Mello S, Abbott R, Radvansky G, Haass M, Tamplin A (2013). Individual differences in multitasking ability and adaptability. Hum Factors.

[CR39] Nashed JY, Crevecoeur F, Scott SH (2014). Rapid Online Selection between Multiple Motor Plans. J Neurosci.

[CR40] Newell A, Simon HA (1972). Human problem solving.

[CR41] Patla AE, Prentice SD, Robinson C, Neufeld J (1991). Visual control of locomotion: strategies for changing direction and for going over obstacles. J Exp Psychol Hum Percept Perform.

[CR42] Pierrieau E, Lepage JF, Bernier PM (2021). Action costs rapidly and automatically interfere with reward-based decision-making in a reaching task. eNeuro.

[CR43] R Core Team. (2019). R: A Language and Environment for Statistical Computing. In R Foundation for Statistical Computing. https://www.R-project.org/. Accessed 22 Sep 2021

[CR44] Raßbach P, Grießbach E, Cañal-Bruland R, Herbort O (2021). Deciding while moving: cognitive interference biases value-based decisions. Acta Psychol.

[CR45] Rowe JB, Siebner HR (2012). The motor system and its disorders. Neuroimage.

[CR46] Schad DJ, Vasishth S, Hohenstein S, Kliegl R (2020). How to capitalize on a priori contrasts in linear (mixed) models: a tutorial. J Mem Lang.

[CR47] Schönbrodt FD, Perugini M (2013). At what sample size do correlations stabilize?. J Res Personal..

[CR48] Schuch S, Philipp AM, Maulitz L, Koch I (2021). On the reliability of behavioral measures of cognitive control: retest reliability of task-inhibition effect, task-preparation effect, Stroop-like interference, and conflict adaptation effect. Psychol Res.

[CR49] Shinners P (2011) Pyame—python game development. Accessed from http://www.pygame.org. Accessed 10 Jan 2022

[CR50] Simon JR, Hinrichs JV, Craft JL (1970). Auditory S-R compatibility: reaction time as a function of ear-hand correspondence and ear-response-location correspondence. J Exp Psychol.

[CR51] Spieser L, Servant M, Hasbroucq T, Burle B (2017). Beyond decision! Motor contribution to speed-accuracy trade-off in decision-making. Psychon Bull Rev.

[CR52] Taylor MJ, Dabnichki P, Strike SC (2005). A three-dimensional biomechanical comparison between turning strategies during the stance phase of walking. Hum Mov Sci.

[CR53] Wagenmakers E-J, Morey RD, Lee MD (2016). Bayesian benefits for the pragmatic researcher. Curr Dir Psychol Sci.

[CR54] Watson JM, Strayer DL (2010). Supertaskers: profiles in extraordinary multitasking ability. Psychon Bull Rev.

[CR55] Wispinski NJ, Gallivan JP, Chapman CS (2020). Models, movements, and minds: bridging the gap between decision making and action. Ann N Y Acad Sci.

