# Supplementary material for: Embodied decision biases: individually stable across different tasks?
Source: Exp Brain Res. 2023 Mar 13;241(4):1053–64. doi: 10.1007/s00221-023-06591-z (PMC10082122; doi:10.1007/s00221-023-06591-z)
Supplement: Supplementary file 1 — Supplementary file1 (PDF 461 KB) [file 221_2023_6591_MOESM1_ESM.pdf]

Supplementary Information

Embodied decision biases – individually stable across different tasks?

Eric Grießbach[1*], Philipp Raßbach[2], Oliver Herbort[2]

& Rouwen Cañal-Bruland[1*]

[1] Department for the Psychology of Human Movement and Sport, Friedrich Schiller University Jena, Germany

[2] Department of Psychology, Julius-Maximilians-Universität Würzburg, Germany

*Correspondence concerning this article should be addressed to Eric Grießbach (eric.griessbach@uni-jena.de) or Rouwen Cañal-Bruland (rouwen.canal.bruland@uni-jena.de).

Funding: This work was supported by the German Research Foundation (DFG) with two grants awarded to RCB (CA 635/4-1) and OH (HE 6710/4-1).

Supplementary Information

Turning while walking task (TWWT)

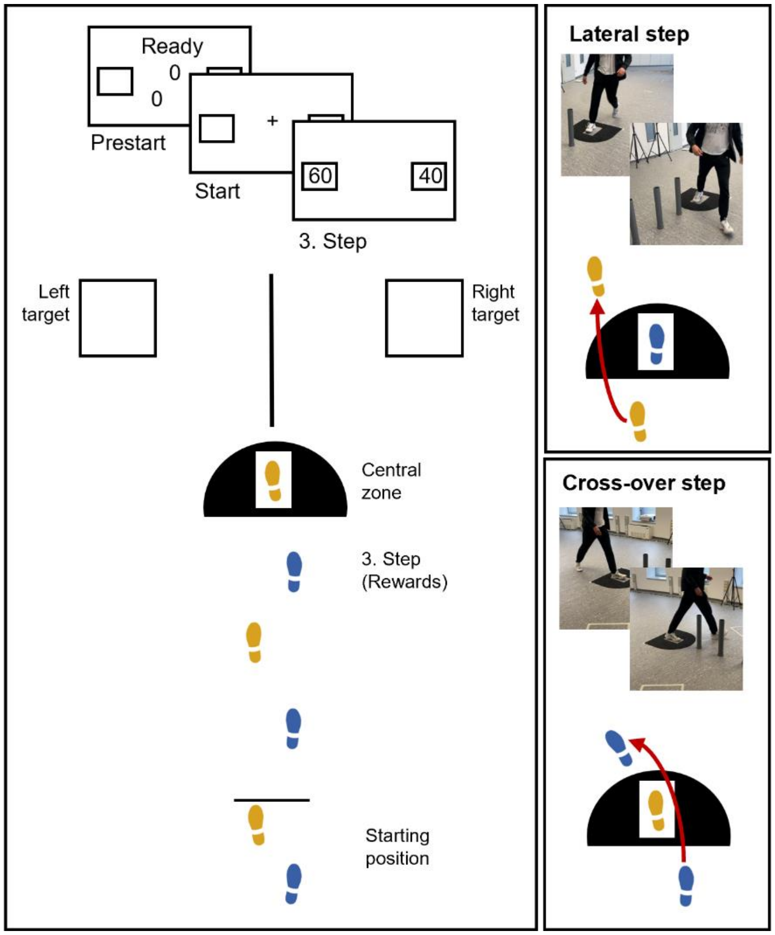

Fig. S1. The Turning While Walking Task (TWWT). Participants started by positioning their feet in the required starting position. The starting position was displayed with a projector on the opposite side of the room (see rectangles at the top). After participants took the required starting position, they walked toward the central zone. After three steps, rewards for the left and right sides were projected on the screen. Participants had to step into the central zone and walk towards a target area to finish a trial and receive rewards. Right side top: Example of a lateral step. Given that the right foot stepped into the zone and participants chose to walk to the left side, a lateral step could be taken. Right side bottom: Example of a cross-over stepping strategy. Given that the right foot stepped into the zone, we assumed participants to make a cross-over step towards the right side.

Calibration and familiarization trials

Similar to our previous study (Grießbach et al., 2022), the starting position and the time constraint, and hence control for task difficulty between participants, were individualized by performing calibration trials before the experiment. In calibration trials, participants had to walk

as fast as possible between both ends of the room for five trials before experimentation. To achieve an average distance of four steps before reaching the central zone, the starting line was shifted so that the distance between the starting line and the midpoint of the central zone was 0.22 m smaller than the mean walking distance of the first four steps in the calibration trials, but maximally 3.41 m because of the length of the room. The time constraint was calculated as the average time to make four steps in the calibration time plus 2.1 s (Grießbach et al., 2022). For calibration trials, we defined the trial start as the first time where one of the lateral malleoli markers exceeded a horizontal velocity of 0.1 m/s for 0.125 s consecutively, based on the difference (derivative) between the position of consecutive frames.

In the following familiarization trials, to familiarize with the time constraint the required time to finish was projected on the screen for the first six trials, and no auditory feedback was given regarding whether participants were too late or too early. In the following six trials and for the rest of the experiment, auditory feedback was provided to indicate whether participants were in time.

Online analysis

Identical to Grießbach et al. (2022), the following conditions had to be met for 180 frames (1.5 s) to start a trial:

1. Six markers had to be in an area around the starting line (-0.3 to 2.1 m horizontal, -0.3 to 0.3 lateral, and under 0.25 m height). If six markers were in the area, these markers were identified by the assumed starting position (left foot positioned left to the right foot, toe positioned more to the front than the lateral malleolus, lateral malleolus more to the front than the heel).

2. The most forward marker had to be close to the starting line (horizontal ± 0.05 m, lateral ± 0.3 m).

3. The predetermined foot had to be in front.

4. The malleolus marker stood still, i.e., was not displaced between consecutive frames for more than 0.004 m.

To present rewards at the third step, the touch-down (first contact of the foot with the ground) of every step was estimated kinematically (Banks et al., 2015). A touch-down was defined when the horizontal distance of the heel marker of the swing leg and the lateral malleolus marker of the stance leg reached a maximum, i.e., the horizontal difference of the position between two frames inverted from positive to negative. To ensure one maximum per touchdown, the analysis of touchdowns was paused for 0.125 after finding a touchdown. To check whether participants stepped into the central zone, we compared the position of the lateral malleolus marker of all touch-downs to the area of the central zone. If the participant did not step into the central zone a warning message appeared centrally on the projected display ("Markierung beachten" in German, freely translated as "Note marking"). A trial ended if more than four markers were in the target area (more than one foot. The time from trial start to the end was measured with MATLABs intern stopwatch timer ("tic", "toc") and used as comparison and feedback to check whether participants finished in time.

Offline analysis

To identify the correct foot stepping into the zone, we checked the kinematic data visually for trials in which:

1. the foot in the mark was not defined by the online analysis,
2. a second self-written velocity-based algorithm for estimating the touch-down did not agree with the distance-based algorithm from Banks et al. (2015),

3. the toe of the foot first passing the beginning of the zone did not agree with the foot touch-down in the zone,
4. rewards were presented too late when the foot was already in the central zone,
5. participants switched sides rapidly (0.25 s) before a trial finished, as an indication of ignoring the obstacle.
6. a trial was not finished, or there was no kinematic data available,

Multilane Tracking Task (MLTT)

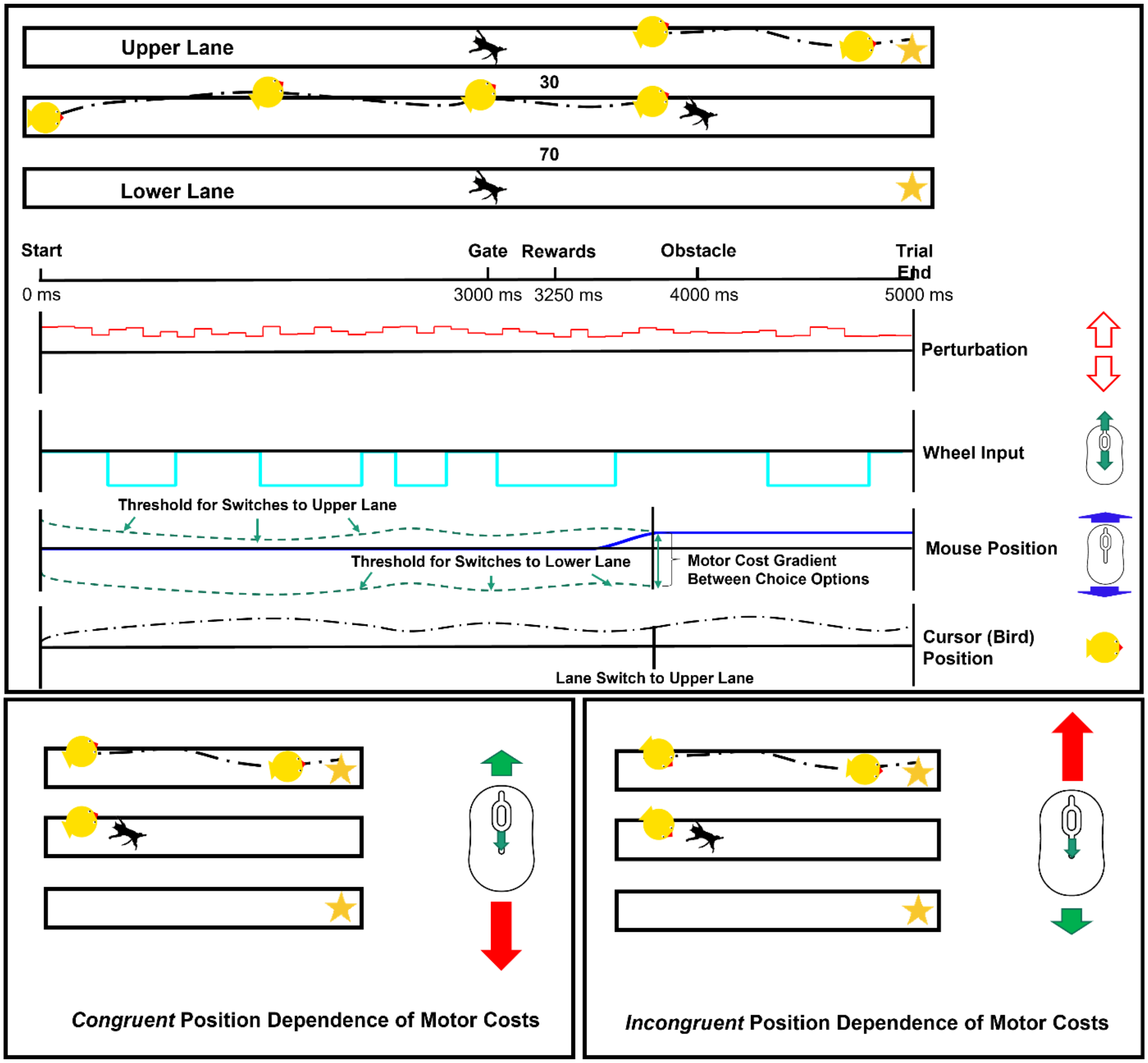

Fig. S2. The Multilane Tracking Task (MLTT). Each trial in the MLTT started with the bird on the middle lane. After 1000 ms, the visual scenery started to scroll leftward, giving the impression that the bird moved rightward. From then on, the perturbation shifted the y-axis position of the bird either upward or downward (upward in the depicted exemplary trial) and participants had to counteract this by scrolling with the mouse wheel either downward or upward. After 3000 ms of tracking, participants passed the gate after which a lane switch could be performed. Rewards and obstacles were visible after 3250 ms, with the point distribution being depicted as numerals above and below the middle lane slightly in front of the cursor. Lane switches were performed by sliding the computer mouse either forward (switch to upper lane) or backward (switch to lower lane). For this, participants had about 750 ms after reward onset until collision with the central obstacle. Depending on the position dependence of motor costs condition, the motor costs for each choice varied as a function of the bird's position. In the shown trial, the congruent condition is depicted (see also lower left panel at the bottom; the incongruent condition is depicted in the lower right panel), with lane switches to the lane the bird was positioned closer to requiring a lower magnitude of mouse movement. Here, a switch to the upper lane is depicted (i.e., a lower-cost switch). This is comparable to participants choosing a lateral step in the TWWT (see fig. S1). The reward on the respective lane was collected automatically. For original video footage, the reader is referred to the online repository of this study.

Determination of the movement threshold in the MLTT

Given the mouse dpi setting of 1200, a constant weight for the position of the bird avatar of 0.36, and a mouse cursor sensitivity setting of $\frac{1}{8}$ (i.e., 3 on the respective Windows cursor sensitivity scale), the necessary movement magnitude for performing a lane switch was determined via the following formula:

$$m = \frac{300\,px \pm d \times \frac{1}{0.36}}{\left(1200 \times \frac{1}{2.54}\right) \times \frac{1}{8}}$$

with *m* being the required movement amplitude (in cm) and *d* being the absolute distance between the center of the bird avatar and the center of the middle lane (in pixels). Note that the movement amplitude for a lane switch to the lane affording lower motor costs referred to subtracting the second term in the numerator from the first term, while a lane switch affording higher motor costs referred to adding the second term in the numerator to the first term. Consequently, if the bird was positioned exactly in the middle of the lane ($d = 0$), a movement of 300 pixels ($m = 6.10$ cm) of the mouse cursor (computer mouse) was necessary to switch to either lane. Note that the required mouse cursor movement was capped by the screen resolution and could not exceed 535 pixels (about 9.06 cm given the setup) in either direction to account for the screen restrictions.

Data Analysis

Moreover, we used weakly informative priors for all parameters. To determine these priors, we relied on prior predictive checks on the probability scale, aiming for a roughly uniform distribution for decisions. For the intercept we used a normal distribution ($\mu = 0$, $\sigma = 1$). For all other regression coefficients, we used a slightly narrower normal distribution ($\mu = 0$, $\sigma =$

0.5). We used an exponential distribution for the standard deviation of all random effects (λ = 1). For the correlation parameter, we used a Lewandowski-Kurowicka-Joe distribution (η = 3). This results in the following formula for the model to be fitted to the data (McElreath, 2019):

$$Right_{i,TWWT} \sim Binomial(n_{i,TWWT}, p_{i,TWWT})$$

$$Right_{i,MLTT} \sim Binomial(n_{i,MLTT}, p_{i,MLTT})$$

where the subscript *i* expresses the individual rows (indexes) in the date frame, the subscript *MLTT* and *TWWT* expresses the correspondence to the individual experiments, *n* expresses the number of trials specific to each condition and participant, *Right* expresses the frequency the participant went towards the right side (vs. left side) and p expresses the probability to go towards the right side which is estimated by the following formula:

$$\begin{bmatrix} logit(p_{i,TWWT}) \\ logit(p_{i,MLTT}) \end{bmatrix} = \begin{bmatrix} \alpha_{TWWT_{Part}} + \beta_{SLE_{Part}} \times SLE \\ \alpha_{MLTT_{Part}} + \beta_{ScrD_{Part}} \times ScrD + \beta_{PosD_{Part}} \times PosD \end{bmatrix}$$

Note that the outcome p is logit transformed to linearize the binomial distribution and is dependent on the experiment specific intercept (α) and independent variables (Swing Leg Effect *SLE*; Scrolling Direction *ScrD*; Position Dependence *PosD*). β indicates the to be determined slope parameter which is multiplied with the respective part of the model matrix (e.g., *SLE*, dependent on the contrast). The subscript *Part* indicates that the parameter is participant dependent. However, as formulized below, instead of fitting every participant individually, mixed models fit only one variance parameter for each parameter. This is formalized by a multivariate normal distribution with a population average, and a covariance matrix down below:

$$\begin{bmatrix} \alpha_{TWWT_{Part}} \\ \beta_{SLE_{Part}} \\ \alpha_{MLTT_{Part}} \\ \beta_{ScrD_{Part}} \\ \beta_{PosD\ Part} \end{bmatrix} = MVNormal\left(\begin{bmatrix} \bar{\alpha}_{TWWT} \\ \bar{\beta}_{SLE} \\ \bar{\alpha}_{MLTT} \\ \bar{\beta}_{ScrD} \\ \bar{\beta}_{PosD} \end{bmatrix}, S\right)$$

$$S = \begin{pmatrix} \sigma_{\alpha_{TWWT}} & 0 & 0 & 0 & 0 \\ 0 & \sigma_{\beta_{SLE}} & 0 & 0 & 0 \\ 0 & 0 & \sigma_{\alpha_{MLTT}} & 0 & 0 \\ 0 & 0 & 0 & \sigma_{\beta_{ScrD}} & 0 \\ 0 & 0 & 0 & 0 & \sigma_{\beta_{PosD}} \end{pmatrix} R \begin{pmatrix} \sigma_{\alpha_{TWWT}} & 0 & 0 & 0 & 0 \\ 0 & \sigma_{\beta_{SLE}} & 0 & 0 & 0 \\ 0 & 0 & \sigma_{\alpha_{MLTT}} & 0 & 0 \\ 0 & 0 & 0 & \sigma_{\beta_{ScrD}} & 0 \\ 0 & 0 & 0 & 0 & \sigma_{\beta_{PosD}} \end{pmatrix}$$

$$R = \begin{pmatrix} 1 & \rho_{\beta_{SLE}:\alpha_{TWWT}} & \rho_{\alpha_{MLTT}:a_{TWTT}} & \rho_{\beta_{ScrD}:\alpha_{TWWT}} & \rho_{\beta_{PosD}:\alpha_{TWWT}} \\ \rho_{\alpha_{TWWT}:\beta_{SLE}} & 1 & \rho_{\alpha_{MLTT}:\beta_{SLE}} & \rho_{\beta_{ScrD}:\beta_{SLE}} & \rho_{\beta_{PosD}:\beta_{SLE}} \\ \rho_{\alpha_{TWWT}:a_{MLTT}} & \rho_{\beta_{SLE}:\alpha_{MLTT}} & 1 & \rho_{\beta_{ScrD}:\alpha_{MLTT}} & \rho_{\beta_{PosD}:a_{MLTT}} \\ \rho_{\alpha_{TWWT}:\beta_{ScrD}} & \rho_{\beta_{SLE}:\beta_{ScrD}} & \rho_{\alpha_{MLTT}:\beta_{ScrD}} & 1 & \rho_{\beta_{PosD}:\beta_{ScrD}} \\ \rho_{\alpha_{TWWT}:\beta_{PosD}} & \rho_{\beta_{SLE}:\beta_{PosD}} & \rho_{\alpha_{MLTT}:\beta_{PosD}} & \rho_{\beta_{ScrD}:\beta_{PosD}} & 1 \end{pmatrix}$$

The overlined parameters (e.g. $\bar{\beta}$) indicates population averages, S is the Covariance matrix here in the form of the product of standard deviations (σ) and the Correlation matrix R with the repective correlations ρ. These are the final parameters to be estimated. The outcome of the model is a posterior distribution for each arameter given the likelihood of the data and prior distributions. Prior distributions for these parameters are specified below:

$$\bar{\alpha}_{TWWT} \sim N(0,1)$$

$$\bar{\beta}_{SLE} \sim N(0, 0.5)$$

$$\bar{\alpha}_{MLTT} \sim N(0, 1)$$

$$\bar{\beta}_{ScrD} \sim N(0, 0.5)$$

$$\bar{\beta}_{PosD} \sim N(0, 0.5)$$

$$\sigma_{\alpha_{TWWT}} \sim Exp(1)$$

$$\sigma_{\beta_{SLE}} \sim Exp(1)$$

$$\sigma_{\alpha_{MLTT}} \sim Exp(1)$$

$$\sigma_{\beta_{ScrD}} \sim Exp(1)$$

$$\sigma_{\beta_{PosD}} \sim Exp(1)$$

$$\rho_{\alpha_{TWWT}:\beta_{SLE}} \sim LKJ(3)$$

$$\rho_{\alpha_{TWWT}:a_{MLTT}} \sim LKJ(3)$$

$$\rho_{\alpha_{TWWT}:\beta_{ScrD}} \sim LKJ(3)$$

$$\rho_{\alpha_{TWWT}:\beta_{PosD}} \sim LKJ(3)$$

$$\rho_{\beta_{SLE}:\alpha_{MLTT}} \sim LKJ(3)$$

$$\rho_{\beta_{SLE}:\beta_{ScrD}} \sim LKJ(3)$$

$$\rho_{\beta_{SLE}:\beta_{PosD}} \sim LKJ(3)$$

$$\rho_{\alpha_{MLTT}:\beta_{ScrD}} \sim LKJ(3)$$

$$\rho_{\alpha_{MLTT}:\beta_{PosD}} \sim LKJ(3)$$

$$\rho_{\beta_{ScrD}:\beta_{PosD}} \sim LKJ(3)$$

N indicates the normal distribution with parameters μ (mean) and σ (standard deviation), *Exp* the exponential distribution with rate parameter λ, and *LKJ* indicates the Lewandowski-Kurowicka-Joe distribution with shape parameter η. For fitting this model, we used the following notation as input for the formula to brms in our R script:

$$f_{TWWT} = bf(logit(Right \mid trials(Trial) + \ subset(TWTT)) \sim$$

$$Intercept + SLE + \left(Intercept + SLE \middle| p \middle| Part\right)\right)$$

$$f_{MLTT} = \ bf(logit(Right \mid trials(Trial) \ + \ subset(MLTT)) \sim$$

$$Intercept + PosD * ScrD + \left(Intercept + PosD * ScrD \middle| p \middle| Part\right)\right)$$

$$formula = f_{TWWT} + f_{MLTT} \ + set_r escor(FALSE)$$

Brms is a high-level interface to fit Bayesian models using Stan (Bürkner, 2017; Stan Development Team, 2019). Stan uses an MCMC algorithm (no-U-turn sampler, NUTS) for sampling the posterior distribution. We sampled twelve independent Markov chains with 8000 samples each. The first 2000 samples were warm-up samples, only the last 6000 samples were used to approximate the posterior distribution. To check whether samples converged, we visually inspected the chains and the Rhat statistic (all values were below 1.01). The effective sample size for all relevant parameters was higher than 10000.

Literature

Banks, J. J., Chang, W. R., Xu, X., & Chang, C. C. (2015). Using horizontal heel displacement to identify heel strike instants in normal gait. *Gait Posture*, *42*(1), 101-103. https://doi.org/10.1016/j.gaitpost.2015.03.015

Bürkner, P.-C. (2017). brms: An R Package for Bayesian Multilevel Models Using Stan. *Journal of Statistical Software*, *80*(1), 1 - 28. https://doi.org/10.18637/jss.v080.i01

Grießbach, E., Raßbach, P., Herbort, O., & Canal-Bruland, R. (2022). Embodied decisions during walking. *Journal of Neurophysiology*, *128*(5), 1207-1223. https://doi.org/10.1152/jn.00149.2022

McElreath, R. (2020). Statistical Rethinking: A Bayesian Course with Examples in R and Stan (2nd ed.). Chapman and Hall/CRC.

Stan Development Team. (2019). *Stan Modeling Language Users Guide and Reference Manual*. In (Version 2.21.0) https://mc-stan.org. https://mc-stan.org
